# Data for modeling the height and diameter of *Pinus merkusii* and *P. michoacana* in Zambia

**DOI:** 10.1016/j.dib.2021.107444

**Published:** 2021-10-02

**Authors:** Phillimon Ng'andwe, Donald Chungu, Frank Tailoka

**Affiliations:** a Copperbelt University, School of Natural Resources, P.O. Box 21692, Kitwe, Zambia; b Copperbelt University, Directorate of Distance Education and Open Learning, P.O. Box 21692, Kitwe, Zambia; c Mukuba University, P.O. Box 20382, Kitwe, Zambia

**Keywords:** Allometry, Pine trees data, Mixed-effect, *h-d* model

## Abstract

Data on tree height and diameter of non-native *Pinus merkusii* (Jung. & de Vriese) and *Pinus michoacana* (Martinez) collected from Zambia's non-native forest plantations is presented here. A total of 1542 and 1883 pairs of height and diameter datasets for developing and testing the models are presented, respectively. During a five-year interval forest inventories, data collected supported the development of allometric relationships between height and diameter for the country's two species. Datasets are intended to be reused for various purposes to enhance the understanding of the tree height and diameter relationships of new and existing planted non-native species in Zambia. For detailed examples of the application of this data, see the article, “Stand characteristics and climate modulate height to diameter relationship in Pinus merkusii and P. michoacana in Zambia” Ng'andwe et al., 2021.

## Specifications Table

**Table:** 

Subject area	Agriculture sciences
More specific subject area	Modeling height and diameter allometric relationships
Type of data	Table, graph, figure
How data was acquired	Data presented were collected during the forest plantation inventories of 2011 and 2016 in Copperbelt province in Zambia. We used the Swedish Haglöf diameter mantax blue calipers to collect diameter data, and for height, we used the Finnish Sunnto clinometers. The Garmin GPS 62s were used for locating stands and sample plots. Microsoft Excel was used for initial data processing, and R studio implemented in the R statistical software version 3.5.1 was used for data exploration and modeling.
Data format	Raw, filtered, analyzed
Parameters for data collection	Inventory crews were trained to use forest plantation maps, GPS, and mensuration equipment and establish sample plots, collecting diameter and height data. Data was collected at a five-year interval from the same stands aged above five years and different temporary sample plots were used for each measurement occasion.
Description of data collection	Height and diameter data were collected from the 50,000-hectare forest plantation during national forest inventories. All the forest stands above five years were assessed using temporary random sample plots. Trained inventory enumerators collected data. Data was captured in Microsoft Excel, cleaned, and saved in CSV format for further analysis
Data source location	Institution: **Copperbelt University** City/Town/Region: **Kitwe, Copperbelt region** Country: **Zambia** Data were collected from the four forest plantation sites, namely Chati (12°50′ S, 27°47′ E, 1290 m above sea level (asl), Ichimpe (12°49′ S, 28°13′ E, 1313 m asl), Lamba (13°00′ S, 27°48′ E, 1303 m asl) and Ndola (12°55′ S, 28°26′ E, 1302 m asl) in the Copperbelt province, Zambia
Data accessibility	All data used and generated are included with this article in the repository Ng'andwe, Phillimon; Chungu, Donald; Tailoka, Frank (2021), “Height and diameter data for Pinus merkusii and P. michoacana in Zambia”, Mendeley Data, V2, https://doi.org/10.17632/2ymx9m5jry.2
Related research article	Ng'andwe, P., Chungu, D. and Tailoka, F., 2021. Stand characteristics and climate modulate height to diameter relationship in *Pinus merkusii* and *P. michoacana* in Zambia. *Agricultural and Forest Meteorology*, *307*, p.108510. https://doi.org/10.1016/j.agrformet.2021.108510

## Value of the Data

• The data presented will enhance the development of allometric relationship models between tree height and diameter of non-native tropical pine plantations.
• The end-use potential for these data include data scientists, Forest managers, Researchers, and Policymakers.
• Data presented is suitable for establishing the height-diameter relationships in new and successive plantations and dendrochronology.
• Further applications include forest stand volume, biomass equations for carbon accounting, developing site index for non-native tropical pines, and machine learning experiments.

## 1 Data Description

*Pinus merkusii* Jungh. & de Vriese and *Pinus michoacana* Martinez; were among the first introduced fast-growing non-native trees planted in Zambia. Therefore, the data presented here include tree height and diameter pairs collected during forest inventories in the Copperbelt province in Zambia. These data were collected from the four different plantation sites (i.e., Chati, Ichimpe, Lamba, and Ndola). We present datasets comprising 1,542 trees (i.e., 761 and 781 trees of *P. merkusii* and *P. michoacana*, respectively). These datasets are summarized in Table 1, Table 2, Table 3, and include number of trees, minimum, median, mean, and maximum values for age, diameter, and height.

**Table 1:** Tree data presented for non-native *merkusii* in Zambia.

Variable	Chati	Ichimpe	Lamba	Ndola	Combined
Number of trees	85	192	183	301	761
Age (years)					
Minimum	28.0	27.0	27.0	26.0	26.0
Median	29.9	31.0	29.0	27.0	29.0
Mean	29.4	31.2	29.3	28.6	29.5
Maximum	33.0	34.0	31.0	33.0	34.0
Diameter (cm)					
Minimum	8.0	6.0	9.0	8.0	6.0
Median	26.0	23.0	25.0	22.0	24.0
Mean	26.5	24.1	24.8	22.6	23.9
Maximum	40	52.0	51.0	40.0	52.0
Height (m)					
Minimum	6.0	6.0	9.0	6.0	6.0
Median	22.0	20.0	22.0	18.0	20.0
Mean	22.3	19.3	21.4	16.9	19.2
Maximum	32.0	30.0	28.0	28.0	32.0

**Table 2:** Tree data presented for non-native *michoacana* in Zambia.

Variable	Chati	Ichimpe	Lamba	Ndola	Combined
Number of trees	54	52	233	442	761
Age (years)					
Minimum	29.0	29.0	29.0	29.0	26.0
Median	29.0	29.0	31.0	31.0	29.0
Mean	29.0	29.0	31.1	30.2	29.5
Maximum	29.0	29.0	34.0	34.0	34.0
Diameter (cm)					
Minimum	11.0	7.0	11.0	10.0	6.0
Median	25.5	24.0	28.0	28.0	24.0
Mean	25.0	24.0	27.6	28.6	24.0
Maximum	42.0	39.0	48.0	50.0	52.0
Height (m)					
Minimum	9.0	8.0	12.0	8.0	6.0
Median	22.0	20.5	23.0	19.0	20.0
Mean	22.7	19.7	22.1	19.4	19.2
Maximum	32.0	24.0	27.0	25.0	32.0

**Table 3:** Aggregated tree data presented for non-native *Pinus merkusii* and *P. michoacana* in Zambia.

Variable	Chati	Ichimpe	Lamba	Ndola	Combined
Number of trees	139	244	416	743	1542
Age (years)					
Minimum	28.0	27.0	27.0	26.0	26.0
Median	29.0	30.0	30.0	29.0	29.0
Mean	29.3	30.7	30.3	29.6	29.9
Maximum	33.0	34.0	34.0	34.0	34.0
Diameter (cm)					
Minimum	8.0	6.0	9.0	8.0	6.0
Median	26.0	23.0	27.0	26.0	26.0
Mean	25.9	24.1	26.4	26.2	25.9
Maximum	42.0	52.0	51.0	50.0	52.0
Height (m)					
Minimum	6.0	6.0	9.0	6.0	6.0
Median	22.0	20.0	23.0	19.0	20.0
Mean	22.4	19.4	21.8	18.4	19.8
Maximum	32.0	30.0	28.0	28.0	32.0

**Supplementary datasets**

dataset: **zamemi_11.csv**

**Fig. 1:**
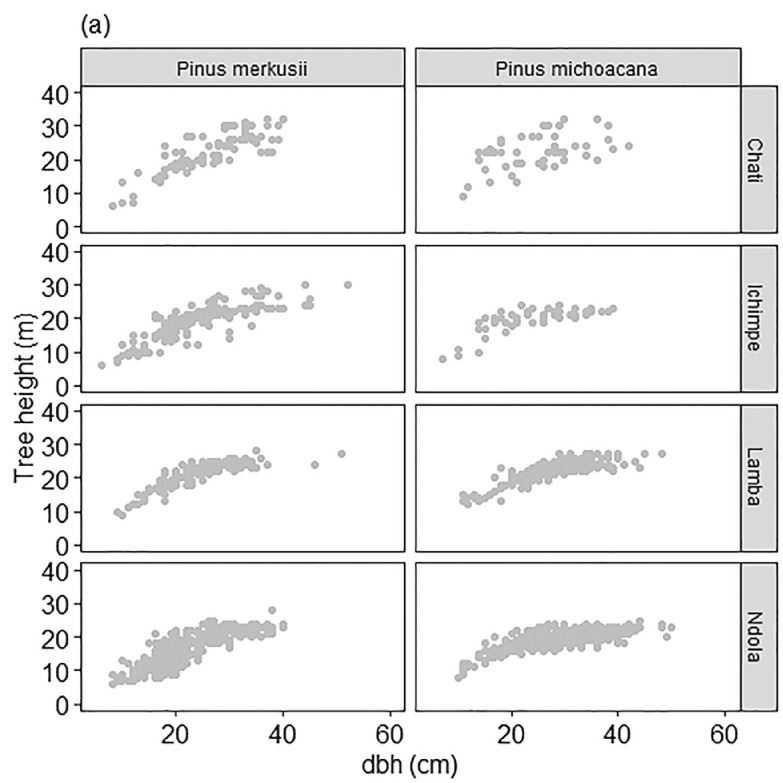
Data visualization of the tree height and diameter relationship in non-native *Pinus merkusii* and *Pinus michoacana* from forest plantations in Zambia. An aggregated dataset in Table 3 was used to generate this plot.

**Fig. 2:**
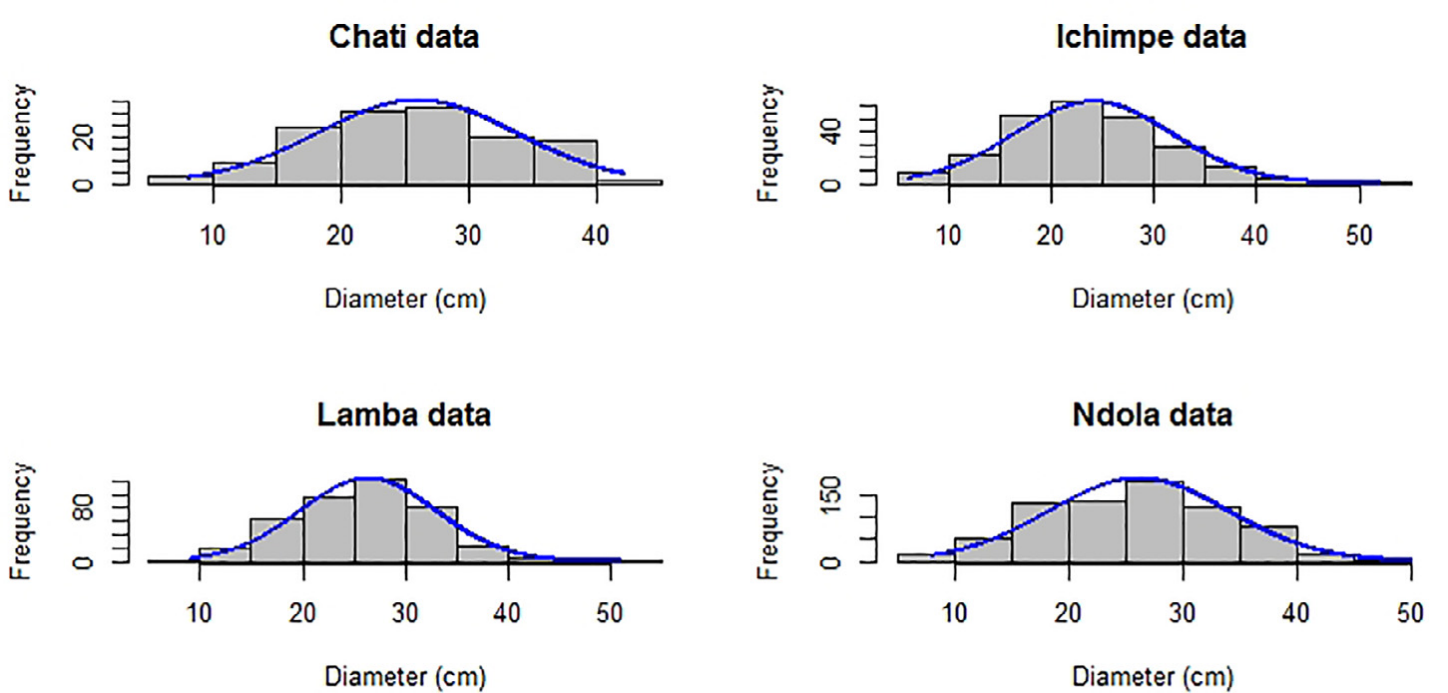
Frequency distribution of diameter at breast height using the aggregated dataset (Table 3) of *Pinus merkusii* and *Pinus michoacana* from non-native forest plantations in Zambia.

This dataset comprises 761 trees with height and diameter pairs for *P. merkussi* and 781 for *P. michoacana,* collected in the Copperbelt Province in Zambia. The data set was used in the present data exploration to generate the descriptive statistics shown in Tables 1, 2 and 3. Height-diameter models were developed from each dataset using the R statistical software [3]. The graphical visualization of this dataset, “zamemi_11.csv”, for model development at each site is presented in Fig. 1 and the frequency distribution of tree diameter in Fig. 2 [4 and 5].

dataset: **zamemi_16.csv**

This file contains 1,883 trees, with height and diameter pairs, for *P. merkusii* and *P. michoacana.* The dataset was used by Ng'andwe et al. [1] to validate height-diameter models for *P. merkusii* and *P. michoacana*. There are 697 and 1,186 trees of *P. merkusii* and *P. michoacana*, respectively. This dataset was collected after five years following the collection of model development data.

## 2 Experimental Design, Materials and Methods

### 2.1 Data acquisition

A probability sampling procedure was applied in determining the sample size and sampling intensity [10] for the 50,000 ha plantation comprising Pine and Eucalyptus species. Factors considered in determining the sample size included the cost, time constraints, and minimum standard errors and personnel. In this regard, an optimum sample size needed to estimate the true population proportion with the required margin of error was determined using Eq. 1.

(1) $$n=\frac{z^{2}*p\left(1-p\right)}{E^{2}}$$

Where:

*n* = optimum sample size.
*z* = value of the standard normal distribution at 95% confidence interval (*z*-value = 1.96).
*p* = expected probability for success.
*E* = margin of error (MOE) between true proportion and the sample proportion, indicating the level of precision required (1-5%).

The margin of error (MOE) was tested between 1-5% using the above formula. The optimum was realized at a 3% margin of error and a sampling intensity of 2.1%. Based on the fixed area random sample plots of 0.05 ha (i.e., 22.4m x 22.4 m), target sample plots were estimated from the sample size (*n*) for *P. merkusii* and *P. michoacana* forest plantation in Copperbelt province.

Sample plots were randomly established in Chati, Ichimpe, Lamba, and Ndola sites. Individual tree data for *P. merkusii* and *P. michoacana* were collected from a network of 1006 sample plots. Tree height (*h*) was measured using the Finnish Sunnto clinometers, and *d* was measured using Haglöf diameter calipers (Swedish Haglöf Mantax Blue). The Garmin GPS 62s was used for locating stands and sample plots. Tree age was obtained from administrative records. Inventory crews were trained to use forest plantation maps, GPS, and mensuration equipment and establish sample plots, collecting diameter and height data [4], [5]. Data was collected at a five-year interval from the same stands aged five years and above and different sample plots for each measurement occasion. All the forest stands above five years were assessed. Data presented were collected during the forest inventories of 2011 and 2016 and include Country, Province, group, species, site, GPS location, stand code, plots, stand age, and measured diameter and total tree height. Data was captured in Microsoft excel, cleaned, and saved in CSV format for further analysis. R studio implemented in the R statistical software version 3.5.1 was used for data exploration and modeling.

### 2.2 Data analysis

**Fig. 3:**
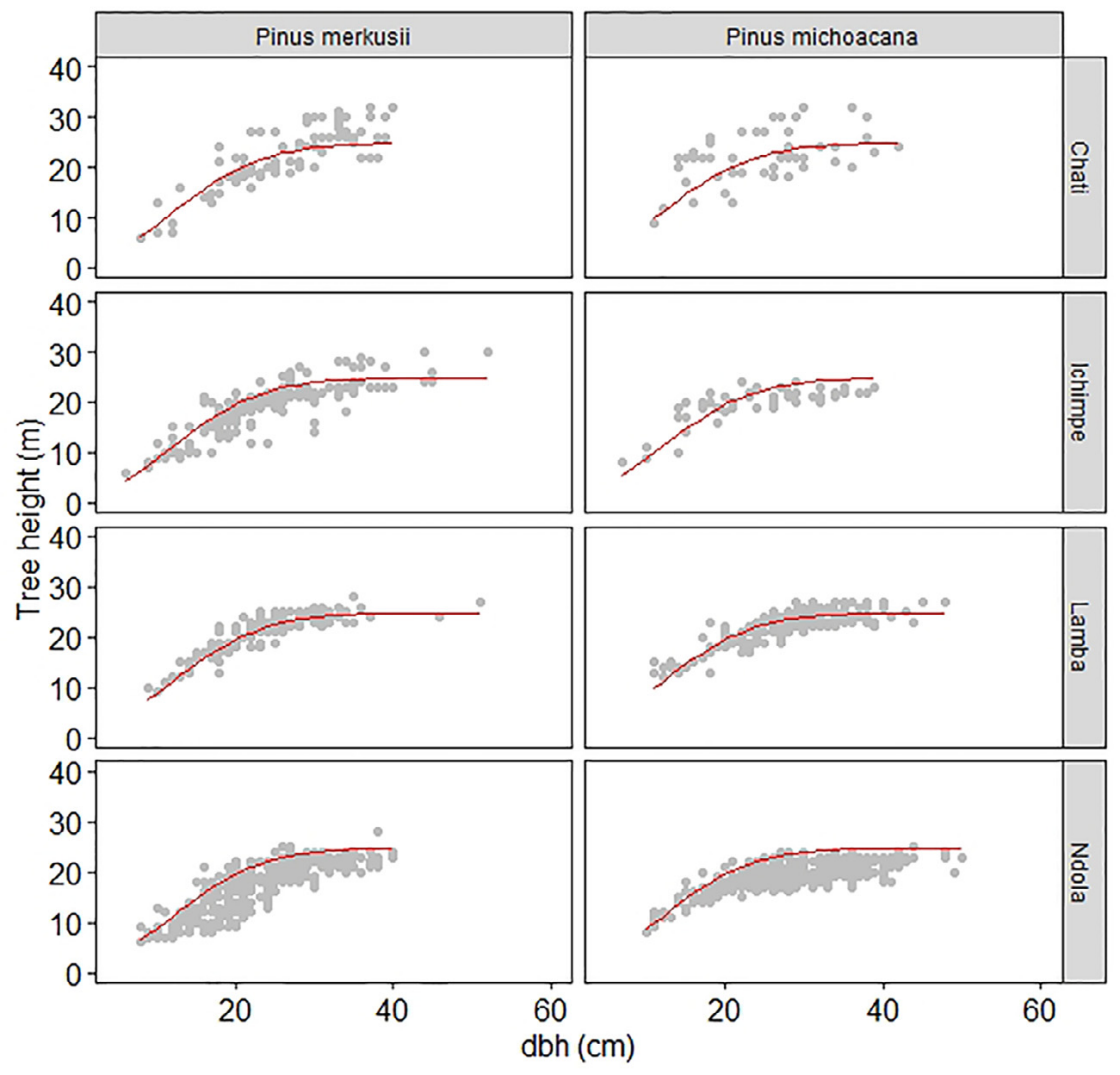
Graphical visualization of model fits to *Pinus merkusii* and *Pinus michoacana* datasets showing scatter plots of height and diameter data overlaid with the curve produced by the country level height-diameter model (Eq. 2). Note that the country-level model fit was used to visualize its performance (i.e acceptable, over or under estimation of tree height of *Pinus merkusii* for each site).

**Fig. 4:**
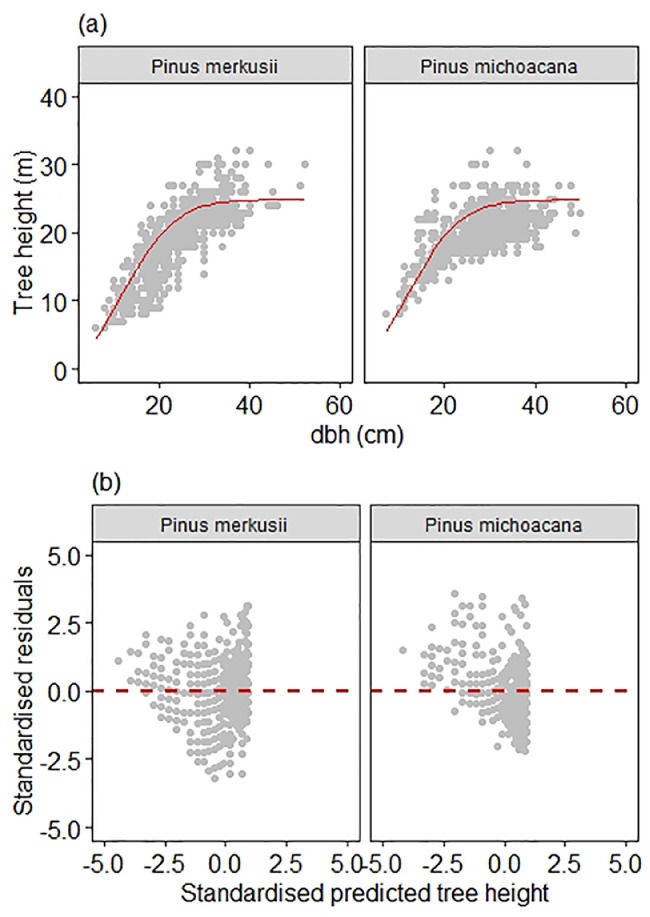
Graphical visualization of model fits to *Pinus merkusii* and *Pinus michoacana* data of (a) scatter plot of height and diameter data overlaid with the curve produced by the country level height-diameter model of the simple fixed-effect model type (Eq. 2), and (b) Standardized residuals vs. standardized predicted height. Note that the country-level model fit produced a megaphone pattern suggesting non-constant variance.

To develop a single model capable of predicting tree height in *P. merkusii* and *P. michoacana,* we pooled datasets into group data (Table 3). We first used the country level *h-d* for *P. kesiya* in Eq. 1 recommended by Ng'andwe et al. [6] as a base model. Eq. 2 was tested on the species dataset and evaluated using the mean absolute percent error (MAPE) (Eq. 3), graphically (Figs. 3 and 4a), and use of residual vs. predicted to check for heteroscedasticity (Fig. 4b).

(2) $$H\;=1.3+23.520\left(1-\text{exp}(-0.0041d^{1.965})\right)$$

 Where *H* is the predicted tree height (m), exp is the exponential, and *d* is the diameter (cm) at breast height.

The country-level height-diameter model (Eq. 2), tested graphically on the site dataset for *P. merkusii* and *P. michoacana,* is shown in Fig. 3.

Testing Eq. 2 to the pooled data was aimed at producing a single model for predicting both species' tree height at the same time. We assessed any striking megaphone patterns of moel fit to the dataset suggesting non-constant variance (Fig. 4).

We next fitted simple fixed-effect *h-d* models on pooled data using the frequently used Curtis, Chapman-Richards, and Weibull theoretical functions based on the non-linear least-squares method. To evaluate the models, we used MAPE (Eq. 3), and the root mean square error (RMSE) (Eq. 4) as measures of accuracy. For the reliability test, we used the overall model prediction accuracy (MPA) (Eq. 5), which combines the mean prediction bias (MPB) (Eq. 6) and residual standard deviation.

(3) $$\text{MAPE}\;=\;\frac{100}{n}\sum_{i=1}^{n}\frac{\left|\left(h_{i}-{\hat{h}}_{i}\right)\right|}{h_{i}}$$

(4) $$\text{RMSE}\;=\sqrt{1/\left(n-k\right)\sum_{i=1}^{n}{\left(h_{i}-{\hat{h}}_{i}\right)}^{2}}$$

(5) $$\text{MPA}=\;MPB^{\;2}+SD^{2}$$

(6) $$\text{MPB}\;=\;\sum_{i=1}^{n}\frac{\left(h_{i}-{\hat{h}}_{i}\right)}{n}$$

 where, MAPE is the mean absolute percent error; $h_{i}$ is the measured tree height for the ith tree; ${\hat{h}}_{i}$ is the predicted tree height for the ith tree; *n* is the number of measured trees; RMSE is the root mean square error, and k is the number of fixed model parameters; MPB is the mean prediction bias; MPA is the model prediction accuracy which combines mean prediction bias and the residual standard deviation.

**Fig. 5:**
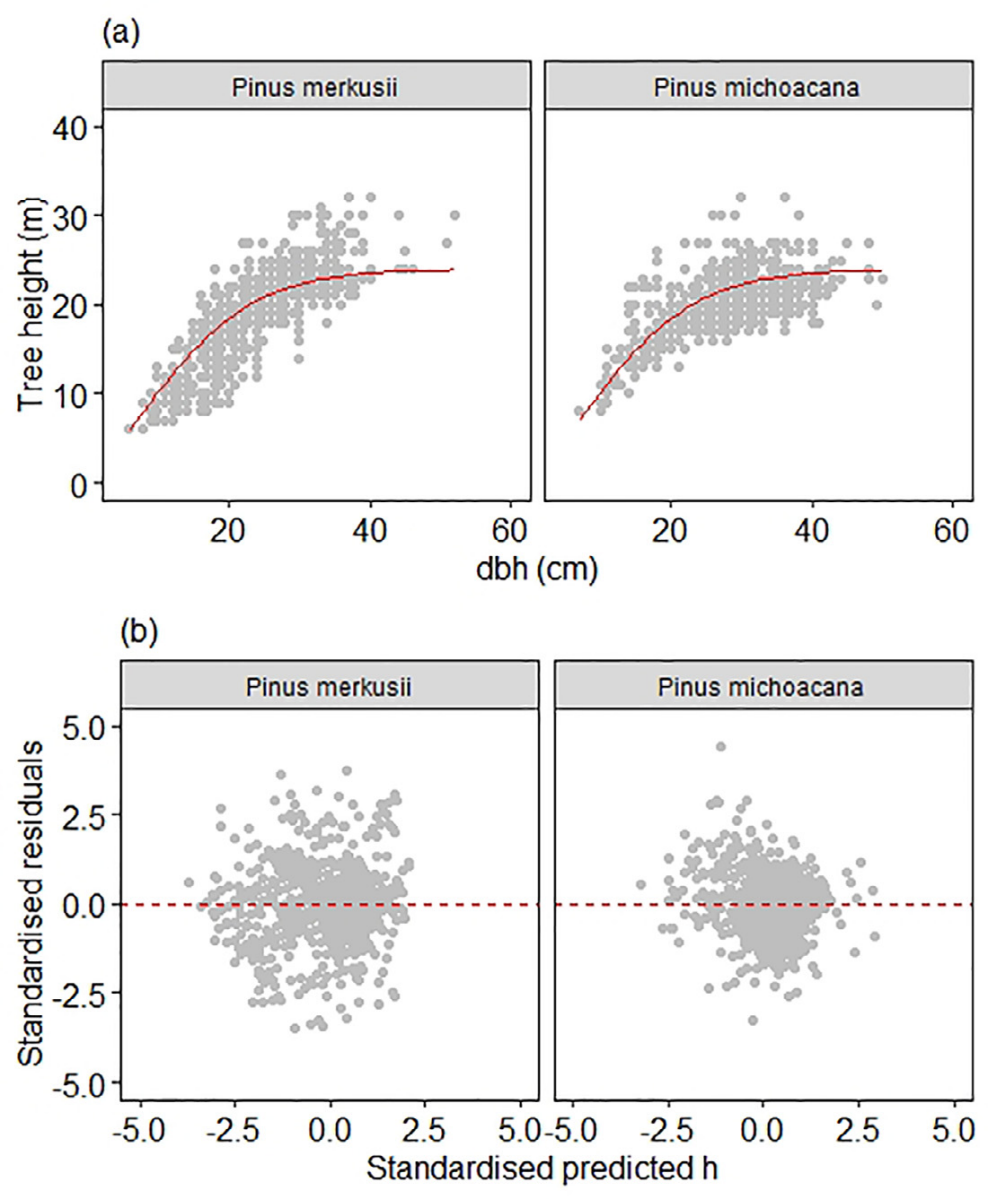
Graphical visualization of model fits to *Pinus merkusii* and *Pinus michoacana* data of (a) scatter plot of height and diameter data overlaid with the curve produced by the simple mixed-effect height-diameter single model, and (b) Standardized residuals vs. standardized predicted height. Note that the simple mixed-effect model's fit produced no striking patterns suggesting a constant variance along with the predicted height in both species.

The best *h-d* model was selected based on low MAPE, RMSE, MPA, and rank position. The model based on the Weibull function was found outstanding among the models developed and was refitted using a simple mixed-effect procedure Eqs. 7 and (8).

Before developing a simple mixed-effect model, we created an object of the groupData class [6] with height as a function of measured diameter, allowing parameter estimates to vary with species nested in stands using the non-linear mixed effect (*nlme*) and the *lmfo*r package [2] in the R statistical software [3]. In this formulation, possible hierarchies (i.e., plots to stands and stands to sites) of the data were taken into account through random effects [2,7]. The model formulation is shown in Eq. 7 for simple fixed-effects and 7 for random effects in the model structure.

(7) $$h_{ij}=f\left(d_{ij,}\beta_{i}\right)+\varepsilon_{ij}$$

 Where *h_ij_* is the total tree height of tree *j*, on plot *i*, where *f* is the best-selected function, *d_ij_* is the diameter at breast height of 1.3 m of tree *j* on plot *i* (i.e., fixed effect term), $\beta_{i}$ is the random effect and $\varepsilon_{ij}$ is the error term, $f(d_{ij,}\beta_{i})$ is the non-linear systematic part of the model.

For the simple mixed-effects model, we allowed the systematic part to vary between plots or stands through the inclusion of random effects as follows:

(8) $$\beta_{i}=B+b_{i}$$

 Where *B* are plot-specific parameters for a typical plot among the population of plots in the stand, and the effect from each plot is denoted by *b*_i_ (the difference between parameters of the typical plot and plot *i*). In this regard, both the fixed and random parameters and their standard errors were estimated simultaneously, and fixed parameters (α, $\beta_{i}$ and γ) of the non-linear model were assumed to vary among levels, as detailed in Mehtätalo et al. [2].

A single model for predicting tree height from measured diameter for *P. merkusii* and *P. michoacana* combined data was developed in this final parameterization. The details of the plot-specific prediction may be obtained from Pinheiro and Bates [7,8], Mehtätӓlo et al. [2], and Lappi [9]. Scatter plot of height and diameter data overlaid with the curve produced by the simple mixed-effect height-diameter single model. Standardized residuals vs. standardized predicted height were used to assess model fit to the aggregated data (Fig. 5a and b). The precision of the simple fixed model and mixed-effect can be compared graphically (i.e. Fig. 4 and Fig. 5, respectively)

## Supplementary Material Information

The data files associated with this article are presented as follows:

1. Filename: zamemi_11.csv
2. Filename: zamemi_16.csv

These supplementary files may be found in the Mendeley repository Ng'andwe, Phillimon; Chungu, Donald; Tailoka, Frank, “Height and diameter data for Pinus merkusii and P. michoacana in Zambia”, Mendeley Data, V2, 10.17632/2ymx9m5jry.2

## CRediT authorship contribution statement

**Phillimon Ng'andwe:** Conceptualization, Investigation, Methodology, Formal analysis, Writing – original draft, Visualization, Data curation. **Donald Chungu:** Software, Project administration, Supervision, Formal analysis, Writing – review & editing, Validation. **Frank Tailoka:** Validation.

## Declaration of Competing Interest

The authors declare that they have no known competing financial interests or personal relationships which have, or could be perceived to have, influenced the work reported in this article.

## Appendix Supplementary materials

Image, application 1

Image, application 2
